# Supplementary material for: Investigating transmission of SARS-CoV-2 using novel face mask sampling: a protocol for an observational prospective study of index cases and their contacts in a congregate setting
Source: BMJ Open. 2022 Nov 23;12(11):e061029. doi: 10.1136/bmjopen-2022-061029 (PMC9684274; doi:10.1136/bmjopen-2022-061029)
Supplement: Author's manuscript [file bmjopen-2022-061029.draft_revisions.pdf]

BMJ Open is committed to open peer review. As part of this commitment we make the peer review history of every article we publish publicly available.

When an article is published we post the peer reviewers' comments and the authors' responses online. We also post the versions of the paper that were used during peer review. These are the versions that the peer review comments apply to.

The versions of the paper that follow are the versions that were submitted during the peer review process. They are not the versions of record or the final published versions. They should not be cited or distributed as the published version of this manuscript.

BMJ Open is an open access journal and the full, final, typeset and author-corrected version of record of the manuscript is available on our site with no access controls, subscription charges or pay-per-view fees (http://bmjopen.bmj.com).

If you have any questions on BMJ Open's open peer review process please email
info.bmjopen@bmj.com

BMJ Open

INVESTIGATING TRANSMISSION OF SARS-COV-2 USING NOVEL FACE MASK SAMPLING: A PROTOCOL FOR AN OBSERVATIONAL PROSPECTIVE STUDY OF INDEX CASES AND THEIR CONTACTS IN A CONGREGANT SETTING

Journal:	*BMJ Open*
Manuscript ID	bmjopen-2022-061029
Article Type:	Protocol
Date Submitted by the Author:	13-Jan-2022
Complete List of Authors:	Jaenisch, Thomas; Colorado School of Public Health, Center for Global Health / Epidemiology; University Hospital Heidelberg, Heidelberg Institute of Global Health<br>Lamb, Molly; Colorado School of Public Health, Center for Global Health / Epidemiology<br>Gallichotte, Emily; Colorado State University, Department of Microbiology, Immunology, and Pathology<br>Adams, Brian; Colorado School of Public Health, Center for Global Health<br>Henry, Charles; Colorado State University, Department of Chemical and Biological Engineering<br>Riess, Jeannine; Colorado State University, Office of Environmental Health Services<br>van Sickle, Joni Triantis; Colorado State University, Office of Environmental Health Services<br>Hawkins, Kellie; Denver Health, Public Health Institute<br>Montague, Brian; University of Colorado, Occupational Health<br>Coburn, Cody; University of Colorado, Occupational Health<br>Conners Bauer, Leisha; University of Colorado Boulder, Health Promotion and Collegiate Recovery Center<br>Kovarik, Jennifer; University of Colorado Boulder, Health Promotion and Collegiate Recovery Center<br>Hernandez, Mark; University of Colorado Boulder, Civil, Environmental and Architectural Engineering<br>Bronson, Amy; Colorado Mesa University, Office of the Vice President<br>Graham, Lucy; Colorado Mesa University, Department of Nursing<br>James, Stephanie ; Regis University, Department of Pharmaceutical Sciences<br>Hanenberg, Stephanie; University of Colorado Colorado Springs, Office of Wellness and Health<br>Kovacs, James; University of Colorado Colorado Springs, Department of Chemistry and Biology<br>Spencer, John; Colorado State University, Department of Microbiology, Immunology, and Pathology<br>Zabel, Mark; Colorado State University, Department of Microbiology, Immunology, and Pathology<br>Fox, Philip; Colorado State University, Department of Microbiology, Immunology, and Pathology<br>Pluss, Olivia; Colorado School of Public Health, Center for Global Health

	Windsor, William; Colorado School of Public Health, Center for Global Health<br>Winstanley, Geoffrey; Colorado School of Public Health, Center for Global Health<br>Olson, Daniel; Colorado School of Public Health, Center for Global Health<br>Barer, Michael; University of Leicester, Department of Infectious Diseases<br>Berman, Stephen; Colorado School of Public Health, Center for Global Health<br>Ebel, Gregory; Colorado State University, Department of Microbiology, Immunology, and Pathology<br>Chu, May; Colorado School of Public Health, Center for Global Health
Keywords:	COVID-19, Diagnostic microbiology < INFECTIOUS DISEASES, VIROLOGY

SCHOLARONE™
Manuscripts

TITLE: INVESTIGATING TRANSMISSION OF SARS-COV-2 USING NOVEL FACE MASK SAMPLING: A PROTOCOL FOR AN OBSERVATIONAL PROSPECTIVE STUDY OF INDEX CASES AND THEIR CONTACTS IN A CONGREGANT SETTING

Authors: Thomas Jaenisch[1,2], Molly M. Lamb[1,2], Emily Gallichotte[3], Brian Adams[1], Charles Henry[4], Jeannine Riess[5], Joni Triantis van Sickle[5], Kellie L. Hawkins[6], Brian Montague[6], Cody Coburn[6], Leisha Conners Bauer[8], Jennifer Kovarik[8], Mark Hernandez[9], Amy Bronson[10], Lucy Graham[11], Stephanie James[12], Stephanie Hanenberg[13], James Kovacs[14], John Spencer[3], Mark Zabel[3], Phil Fox[3], Olivia Pluss[1], William J. Windsor[1], Geoffrey Winstanley[1], Daniel Olson[1], Michael Barer[15], Stephen Berman[1], Gregory Ebel[3], May Chu[1,2]

Affiliations:

[1] Center for Global Health, Colorado School of Public Health, Aurora, CO
[2] Department of Epidemiology, Colorado School of Public Health, Aurora, CO
[3] Department of Microbiology, Immunology, and Pathology, Colorado State University, Fort Collins, CO
[4] Department of Chemical and Biological Engineering, Colorado State University, Fort Collins, CO
[5] Office of Environmental Health Services, Colorado State University, Fort Collins, CO
[6] Public Health Institute at Denver Health, Denver, CO
[7] University of Colorado Anschutz, Occupational Health, Anschutz Medical Center, Aurora, CO
[8] Health Promotion and Collegiate Recovery Center, University of Colorado Boulder, Boulder, CO
[9] Civil, Environmental and Architectural Engineering, University of Colorado Boulder, Boulder CO
[10] Office of the Vice President, Colorado Mesa University, Grand Junction, CO
[11] Department of Nursing, Colorado Mesa University, Grand Junction, CO
[12] Department of Pharmaceutical Sciences, School of Pharmacy, Regis University
[13] Office of Wellness and Health, University of Colorado-Colorado Springs, Colorado Springs, CO
[14] Department of Chemistry and Biology, University of Colorado-Colorado Springs, Colorado Springs, CO
[15] Department of Infectious Diseases, University of Leicester, Leicester, United Kingdom

Corresponding Author:
Thomas Jaenisch, MD PhD
Associate Professor, Center for Global Health & Department of Epidemiology,
Colorado School of Public Health
Aurora, CO 80045
thomas.jaenisch@cuanschutz.edu

Key Words: SARS-CoV-2, transmissibility, transmission, face mask sampling, detection, diagnosis, polyvinyl alcohol, congregant setting, college students, COVID-19, study design

For peer review only - http://bmjopen.bmj.com/site/about/guidelines.xhtml

Abstract:

Introduction: This study aims to measure how transmission of SARS-CoV-2 occurs in communities and to identify conditions that lend to increased transmission focusing on congregant situations. We will measure SARS-CoV-2 in exhaled breath of asymptomatic and symptomatic persons using face mask sampling – a non-invasive method for SARS-CoV-2 detection in exhaled air. We aim to detect transmission clusters and identify risk factors for SARS-CoV-2 transmission in pre-symptomatic, asymptomatic, and symptomatic individuals.

Methods and analysis: In this observational prospective study with daily follow-up, index cases and their respective contacts are identified at each participating institution. Contact definitions are based on CDC and local health department guidelines. Participants will wear masks with polyvinyl alcohol test strips adhered to the inside for 2 hours daily. The strips are applied to all masks used over at least 7 days. In addition, self-administered nasal swabs and (optional) finger prick blood samples are performed by participants. Samples are tested by standard PCR protocols and by novel antigen tests.

Ethics and dissemination: This study was approved by the Colorado Multiple Institutions Review Board (COMIRB) and the World Health Organization Ethics Review Committee and registered with ClinicalTrials.gov (#NCT05145803). From the data generated we will analyze transmission clusters and risk factors for transmission of SARS-CoV-2 in congregant settings. The kinetics of asymptomatic transmission and the evaluation of non-invasive tools for detection of transmissibility are of crucial importance for the development of more targeted control interventions – and ultimately to assist with keeping congregant settings open that are essential for our social fabric.

Strengths and limitations of this study:

- The study employs a novel and non-invasive sampling device
- Following index cases as well as their contacts prospectively enables us to study transmissibility
- Results from the novel sampling device in the face masks are compared with standard nose swab sampling
- The manuscript serves as a template for similar studies to be replicated

For peer review only - http://bmjopen.bmj.com/site/about/guidelines.xhtml

INTRODUCTION

There is an urgent need to better describe and analyze the transmission of SARS-CoV-2 in asymptomatic and pre-symptomatic (infected) individuals, especially regarding the heterogeneity of transmissibility ("super-spreader events"), and within the context of congregant situations. An innovative tool with high sensitivity and minimal invasiveness is needed to obtain the necessary information to fill the current knowledge gaps.

A sizable proportion of individuals infected with SARS-CoV-2 are asymptomatic. These individuals contribute to transmission and are also less likely to get tested. Asymptomatic SARS-CoV-2-infected individuals carry viral loads in conventional samples from the nasopharynx that are in the same range as those of symptomatic individuals, and a significant proportion of SARS-CoV-2 spread is due to asymptomatic individuals [1-6]. An analytical model published by the Centers for Disease Control and Prevention (CDC) found that up to 59% of all transmissions likely resulted from asymptomatic transmission. The model further delineated that 35% of these 59% of infections resulted from those that eventually became symptomatic, while 24% remained asymptomatic [7]. This study also found that the asymptomatic group positive for SARS-CoV-2 was only 25% less infectious compared to those that were symptomatic. Of the current multiple studies on asymptomatic transmission, only 2 studies are related to congregant situations [2 4] and none were designed specifically to study transmission in congregant situations, including index cases and their close contacts.

With the emergence of virus variants with the potential for greater infectivity, there is also a need to monitor viral variants in congregant situations among asymptomatic individuals, and to rapidly provide new information to public health authorities [8]. This is especially true for breakthrough infections that require special monitoring regarding the details of the vaccines (type, intervals, etc.) as well as the virus variants that caused the breakthrough infection [9].

The reference method for detection of SARS-CoV-2 is the reverse transcriptase polymerase chain reaction (RT-PCR), a technology that detects viral RNA from respiratory specimens (sputum or nasal swab) or from saliva. For diagnostic purposes, these samples are usually collected at a single point in time.

For peer review only - http://bmjopen.bmj.com/site/about/guidelines.xhtml

To study the dynamics of SARS-CoV-2 transmission over time, we investigate a non-invasive face mask sampling (FMS) method, where polyvinyl alcohol (PVA) strips are embedded in masks for the detection of SARS-CoV-2 in exhaled breath (Figure 1). This enables us to extend the sampling interval over a time window (hours) and include different activities or risk factors such as singing, shouting, sneezing, or coughing as part of the data collection process.

Figure 1: Picture showing two PVA test strips adhered to the inside of a face mask. (Photo credit: Dr. Michael Barer)

The overall aim of this study is to analyze transmission clusters of SARS-CoV-2 in communities and identify factors that may increase transmissibility, with a focus on congregant situations. We will compare the results from the FMS with the standard nose swab. Within sub-studies, we are interested in better understanding antibody profiles and their correlation with symptom status and transmissibility, as well as the effect of different activities (like singing or exercising) on test positivity in the masks. We also plan follow-up studies (which will need additional ethical approval) investigating long-term outcomes in the college student-age population regarding the incidence and the risk factors associated with Long COVID. Details of the study objectives can be found in Box 1.

BOX 1: Objectives

Study Identifier: clinicaltrial.gov identifier NCT05145803

Objective 1: Identification of risk factors for, and rate of, infection in index cases and for transmission from index case to contacts.

Objective 1.1: Describe demographics and evaluate **risk factors for infection** in index cases

Objective 1.2: Evaluate **risk factors for transmission from index case to contacts (and their contacts)**, including viral load, clinical symptoms, vaccination status, activity and environmental metrics and immune response profiles (for example neutralizing antibodies, cytokine profiles – using a blood sample taken after recovery)

For peer review only - http://bmjopen.bmj.com/site/about/guidelines.xhtml

Objective 1.3: Analyze the **difference in the clinical status (symptomatic vs. asymptomatic)** for index cases as well as secondary cases **by humoral and T-cell immune response profile** (using the blood samples collected).

Objective 1.4: Determine **rate of and time to transmission**, stratified by a variety of risk factors (including presence of clinical symptoms, activity and environmental metrics both in index cases and secondary contacts), analyzing pre-symptomatic and asymptomatic exhalation of SARS-CoV-2 in contacts of index cases.

Objective 2: In confirmed index cases and their contacts, quantify and determine the efficacy of the PVA test strip method for SARS-CoV-2 detection, comparing with the respective 'reference standard' (e.g., nasal swab) collection method, and correlating with the presence of antibodies.

Objective 2.1: Assess the **association between molecular detection by PVA test strip and molecular or antigen detection / quantification** by initial reference diagnostic collection method.

Objective 2.2: Assess the **association between molecular detection by PVA test strip and molecular or antigen detection method** (self-sampling) over the sampling / mask wearing period.

Objective 3: Due to the concern around emerging variants with higher transmissibility, carry out deep sequencing of PCR-positive samples (swabs as well as strips from masks) to derive the genotype of infecting virus.

Objective 3.1: Share in timely fashion the results of the sequencing with the national / regional surveillance system.

Objective 3.2: Assess the **genetic similarities and differences of the SARS-CoV-2 variants** by sequence comparison and by virological culture to assess cell-based infectivity of the strains.

Objective 3.3: Determine the chain of transmission and relationship to clinical phenotype in the index case and contacts where viral RNA or culture permit.

Objective 3.4: Analyze the **difference in rate of transmission** (see also Aim 1) **comparing different SARS-CoV-2 variants**.

METHODS AND ANALYSIS

For peer review only - http://bmjopen.bmj.com/site/about/guidelines.xhtml

Study Design: This is a prospective study with daily follow-up to capture early transmission in the pre-symptomatic phase. Index cases are identified by the routine testing system implemented at each participating institution and contacts of index cases are identified by the site-specific contact tracing system. The contact definition is based on CDC and the local health department guidelines (i.e., individuals who are cohabitating or who were in close [<6 feet], extended [>15 min.] contact with the index case and within 48 hours of diagnosis). Study sites have an ongoing active case detection and contact tracing program. Inclusion criteria and endpoints are described in Box 2.

Study population: This study started in the first quarter of 2021 and is currently under way in college students in Colorado, USA, including University of Colorado (Denver, Boulder, Colorado Springs, Aurora), Colorado State University (Fort Collins), Regis University (Denver), as well as Colorado Mesa University (Grand Junction). We envisage enrolment to be completed by April 2022 but would potentially continue beyond this if high SARS-CoV-2 transmission is documented. Students as well as faculty (and their family) are eligible to be enrolled.

The Face Mask Sampling Method: The FMS method has been successfully used to detect Mycobacterium in breath samples and recently adapted for COVID-19 testing [10]. The sensitivity compared to regular nasal swab sampling has not been conclusively determined (which is part of this research project). The (preliminary) analytical sensitivity of this method was estimated around $\geq 10^4$ virus particles, which corresponds to a cycle threshold (Ct) value of 18-20 in the PCR and is in agreement with recent publications and sample testing by other colleagues at the University of Alberta, Canada [11].

Face mask selection: Commercial 3-layer spunbond polypropylene, tested for breathability (95% filtration) KN95 masks that are OSHA/ FDA approved masks will be provided to the participant. Each mask will be uniquely coded for tracking.

Participant and public involvement: Participants were involved in the selection of best fitting masks in a pilot phase. Feedback was elicited regarding the acceptability and fit of different mask designs.

For peer review only - http://bmjopen.bmj.com/site/about/guidelines.xhtml

PVA test strips: PVA test strips are provided by University of Leicester (UL) under a material transfer agreement and strips will be adhered to pre-determined positions inside the face masks. PVA has a very good safety profile, being already used in cosmetic products (face masque) and is approved as hypo-allergenic and non-toxic for humans [12]. PVA has been used in diagnostic tests [13-15] as well as medical device implant for orthopedic surgery [16] and for theranostic nanoplatform therapeutic imagery [17].

Sample Size: This is an exploratory study. We aim for a up to 3,300 individuals, including participants with confirmed SARS-CoV2 infection (index cases) and their contacts. These estimates are based on the conservative estimate of 5% transmission from index cases to a close contact. With a maximum of 300 index cases and ~10 contacts per index case, we estimate that we would detect ~150 secondary cases in contacts.

Box 2: Key Study Design Information

Inclusion / exclusion

Inclusion Criteria:

1. INDEX PATIENTS: Individuals aged ≥ 18 years of any gender tested positive for SARS-CoV-2 by a molecular reference test (PCR test or antigen test).
2. CONTACTS: Individuals aged ≥ 18 years and known to be contacts of the INDEX PATIENTS

Exclusion Criteria: < age 18 years old, unable to wear a face mask due to underlying condition, not able to consent, pregnancy. In addition, signs of severe disease present at time of enrollment (e.g. difficulty breathing, pain when breathing, tightness of chest or developing irregular heartbeat).

Endpoints

A. Outcome Measure(s):

Objective 1 Outcomes: PVA strip positivity; nose swab test positivity

Objective 2 Outcomes: PVA strip positivity and virus quantification (by PCR); nose swab test positivity; antigen test positivity; presence of antibodies in the blood.

For peer review only - http://bmjopen.bmj.com/site/about/guidelines.xhtml

Objective 3 Outcome: Deep sequencing of PCR-positive samples to derive the genotype of the infecting virus.

Schedule of wearing masks: Participants will be asked to wear a mask with 2 PVA test strips adhered to the inside surface for 2 hours per day. Index cases will complete 7 days of mask wearing and if they test positive by the end of the 7-day period, they will be asked to continue for another 7 days up to day 14. Contacts will complete 7 days of mask wearing as long as they test negative. If testing positive in the first 7 days, they will complete an additional 7 days of mask wearing starting with the day when they first test positive. If by the end of this period they still test positive, the duration will be extended by 2-day intervals with their permission until two consecutive masks wear dates test negative (Figure 2). Upon completion of 2 hours of wear by the participant, the face masks will be placed in a sealed plastic bag, collected daily, and brought into the laboratory where the PVA strips will be put into tubes with Viral Transport Medium (VTM). The VTM tubes with the PVA strips will be eluted and tested by PCR.

Self-administered nasal swabs: We ask the participants to collect a self-administered nasal swab daily by placing a nasal swab approximately 0.5 inches into one nostril and swabbing the inside of the nose. The swab is then immediately placed into a 15ml conical tube containing 2ml of VTM. Samples are put into a separate sealed bag and stored at 4°C until testing.

Questionnaires: An electronic questionnaire is administered at the beginning of the mask wearing period to collect basic demographics (age, gender) and to assess COVID-19 symptoms and activities (including the day when symptoms started). During the follow-up period, daily questionnaires are administered, and we have built in warning signs for potential severe COVID-19 that trigger an auto-alert, in which case we will pro-actively contact the participant and a) ask the participant to stop wearing the masks, and b) advise the participant to seek medical care immediately. We also included an optional assessment of activities like singing or exercising for index cases only (confirmed SARS-CoV-2 positive individuals) on one day using a separate second mask. In the future, the assessment of vital signs or specific symptoms could be added via wearable technologies or app-based recording (see for example https://www.hyfeapp.com/).

For peer review only
For peer review only - http://bmjopen.bmj.com/site/about/guidelines.xhtml

<u>*Blood sampling:*</u> We will ask the participants to conduct self-sampling of blood using dried blood spots from finger pricks [18] every third day (optional - filter papers and self-sealing bags provided along with the daily masks). Antibodies will be eluted from the filter paper to be used for serological investigations. These samples are going to be stored in separate sealed bags and picked up together with the other materials. After recovery, e.g., between day 14 and 56 after quarantine/self-isolation, the participants are invited to return to the clinic for a venous blood sample (EDTA 20-30 ml). The blood is collected to investigate humoral, and T-cell based immune responses including cytokine profiles and other T cell phenotypic surface markers that may be associated with transmissibility and clinical phenotype (e.g., protection versus Long Covid).

Figure 2. PVA Mask Strip study, sampling plan for index case and contact

<u>Laboratory methods:</u> In a biosafety cabinet, PVA strips are removed from inside of the face masks, by cutting them at the ends where they are adhered using double-sided tape. Each strip is placed into a 15ml conical tube containing 2ml of viral transport media (VTM) and incubated at room temperature for at least 30 minutes to allow PVA to dissolve. Samples are stored at 4°C until testing. After collection and elution of the strip, testing will be carried out with the following tests in order to validate the results from the FMS against the reference test:

a) First line: PCR from the eluate of the strip
b) Second line: antigen test, which gives a positive/negative/indeterminate response
c) Third line: virus culture to determine if virus is infectious.

In brief, for PCR testing, two approaches are used. In the first approach, samples (PVA strips and nasal swabs) are briefly vortexed to thoroughly mix. Viral RNA is extracted from 200ul of sample using the Mag-Bind® Viral DNA/RNA 96 Kit following manufacturers protocol. Quantitative real-team PCR (qRT-PCR) is performed using CDC 2019-nCoV primer probes and EXPRESS qPCR Supermix and SuperScript. Samples are considered positive if their N1 cycle threshold (CT) is less than 38. In the second approach, samples (PVA strips and nasal swabs) are briefly vortexed to thoroughly mix. Viral RNA is extracted from 45uL of sample by digesting with 5uL of Proteinase K (PK, NEB) for 15 minutes at 60°C. PK is inactivated for 5 minutes at 95°C. Reverse transcription droplet digital PCR (RT-ddPCR) was performed on PK digested sample using CDC

For peer review only - http://bmjopen.bmj.com/site/about/guidelines.xhtml

2019-nCoV primer probes and One-Step RT-ddPCR Advanced Kit for Probes (Bio Rad). Samples are considered positive if their N1 concentration is > 0.25 copies/uL. The RNA extracted from the FMS and potentially recovered live viruses will be used as basis for deep sequencing [3].

One of our aims is to validate a novel antigen assay for testing PVA strips from the masks. For this assay, electrodes were prepared according to Samper et al. [19]. For sample analysis, 20 µL of PVA mask sample or virus standard was added to the functionalized electrode and incubated for 40 min, and then rinsed with PBST and PBS using solid stream spray bottles. Virus standards were inactivated with 0.1% Igepal and diluted in Hanks Balanced Buffer Solution (HBSS) with 0.1% Tween80 and 0.1% Igepal. Following sample incubation, 20 µL of a 0.5 µg/mL HRP-antibody solution was added and incubated for 25 min, and then rinsed with PBST and PBS using solid stream spray bottles. For measurement, 50 µL of 3,3',5,5'-tetramethylbenzidine (TMB) was added to the electrode and incubated for 2 min. After 2 min, chronoamperometry was run at 0.0 V for 2 min.

For peer review only

Contactless delivery and pick-up of supplies: All masks and other samples are delivered / picked up at the residence of the participants and placed in their own sealed bag with a unique identifier. All exposed masks and collected sample kits are individually packaged compliant with UN3314 packaging safety requirements. For the decontamination of surfaces and PPE, we will use 70% alcohol for immediate decontamination of exposed surfaces, especially those that the participant may have touched. For longer term decontamination, we will use 10 uM methylene blue spray that allows for up to 10 hours of decontamination in light [20]. Hand hygiene solutions will be used to clean hands at the end of each drop-off/pick up activity.

Data management: All study data will be collected using Research Electronic Data Capture (REDCap®). Data cleaning and quality control assessments will be conducted on the REDCap survey data, and a weekly data dashboard will be produced to support the study team in detecting issues with data collection. Indicators of severe disease will be monitored daily. Test results from the reference test, the additional molecular tests, antibody tests, and the PVA strip test will be linked to survey data using a unique anonymized study identification number, and data will be stored on a secure internal server with restricted access at the University of Colorado with no

For peer review only - http://bmjopen.bmj.com/site/about/guidelines.xhtml

personal identifiers. The study analyst will hold the key to the personal identifiers and will unblind results as necessary to inform the institution of any previously unknown positive cases, so that they can follow the institution's protocols regarding informing the case, contact tracing, and quarantine. All data will be cleaned, merged and analyzed using SAS version 9.4 (Cary, NC), to ensure replicability of the process and analysis.

Data analysis: For objective 1, we will first conduct a descriptive analysis of the index cases. Then we will conduct a risk factor analysis using multivariable multi-level regression models to determine symptoms and behaviors that are associated with greater exhalation of SARS-CoV_2 virus, as detected by PVA test strips. We will also analyze presence and timing of symptoms in contacts to determine if PVA strips can detect SARS-CoV-2 in exhaled breath of pre-symptomatic and asymptomatic individuals. We will map the transmission dynamics visually, determine the length of exhalation of SARS-CoV-2 in cases, and calculate the difference in rate of transmission by vaccination status. Using the convalescent blood sample, we will analyze different immune response profiles (neutralizing antibodies, T-cell based assays) stratified by clinical phenotype (symptomatic/asymptomatic, potentially using a symptom score for increased granularity).

To address objective 2, we will calculate the sensitivity and specificity of the FMS method, using the initial reference diagnostic method of the site in index cases. We will then calculate the sensitivity and specificity of the PVA test strips using the concurrently collected nasal swab as the reference standard for both index cases and their contacts. We will also correlate strip test positivity with Ct value of the PCR test (or viral load in a subset) over time and with presence of antibodies (IgA and IgG) over time.

To address the concern about emergence of new virus variants, in objective 3, the FMS eluted materials, original specimen and/or recovered live virus isolates will be subjected to deep sequencing to monitor genetic variants in the study population. We will use these data, when available, to calculate the infectiousness of each viral variant, and to map the transmission chains and association with disease in contacts. We will provide the sequence information of identified SARS-CoV-2 variants (and aliquot of material) to national and global data warehouses for public health surveillance of virus variation that may impact disease transmission and resistance to vaccines. The remaining materials will be stored for longer term so that they can be accessed for public health surveillance purposes.

For peer review only - http://bmjopen.bmj.com/site/about/guidelines.xhtml

ETHICS AND DISSEMINATION

Ethical approval for a multicentric study in adults (college students and faculty) was obtained by the Colorado Multiple Institutional Review Board (COMIRB) at Anschutz Medical Campus in Aurora (#21-2823) and by the World Health Organization Ethics Review Committee (CERC.0096). In addition, ethical approval for a pilot study in middle schools (not described in detail in this manuscript) was obtained by the Colorado Medical Institutional Review Board (COMIRB, #21-4944) and an adapted protocol for elementary schools is prepared. Here we will concentrate on the study in the adult population.

There is only minimal risk to the participant associated with this study. Discomfort may arise form wearing masks for extended periods of time or from additional reference validation tests (optional, nasal swabs, sputum tests and/or blood samples), though these are routine procedures for SARS-CoV-2 protection, testing and surveillance in the partnering sites / institutions. For validation purposes, participants will also carry out self-sampling of the anterior nose, which is a minimal risk procedure, and does not cause a lot of inconvenience (different from the naso-pharyngeal sampling). For comparison purposes, participants may also be asked to provide a saliva sample, which is not associated with any risk and minimal inconvenience. One venous blood sample (20-30ml) will be taken after recovery to investigate humoral, and T-cell based immune responses, including cytokine profiles and other T cell phenotypic surface markers that may be associated with transmissibility and clinical phenotype (protection versus long term Covid). This is a minimal risk procedure, carried out by professionals. Inconvenience includes possible bruising and some pain. Finger prick blood samples are optional and are considered minimal risk. They might be inconvenient, and we will let participants decide about participating in this.

We present this study design as a tool to further investigate the transmissibility of SARS-CoV-2. We want to share our scientific approach implemented in institutes of higher education which have routine COVID-19 monitoring in place. We would be happy to help replicating this study in other settings worldwide. This study will help determine transmission chains and risk factors for transmission of SARS-CoV-2 in congregant settings. PVA test strips, a non-invasive method, are investigated as a novel and promising tool to detect exhalation of SARS-CoV-2 from people who are actively infected as well as their exposed contacts.

For peer review only

For peer review only - http://bmjopen.bmj.com/site/about/guidelines.xhtml

FUNDING STATEMENT

The authors acknowledge that funds for the project were provided by WHO based on a grant from the German Federal Ministry of Health (BMG).

AUTHORS CONTRIBUTIONS

TJ, MML and MC conceptualized the study. MC acquired funding. MB developed the face mask sampling method. MML and WJW developed the data base, curate the data, and developed the data analysis approach. MC, GE, EG, CH, MH, JS, MZ, JK, PF carry out lab investigations and developed the laboratory testing methodology. SB, TJ, GW, BA, KLH, JvS, JR, BM, CC, LCB, JK, OP, WJW, DO developed the participant recruitment strategy and conduct the recruitment. TJ and MC wrote the first draft. All authors contributed to the writing of the manuscript and approved the final version.

COMPETING INTERESTS STATEMENT

TJ, MML, JW, BA, GE, MC report grant support from WHO for this study for salaries or equipment/reagents (see also acknowledgements). SB, OP, WJW, AB, CH, JR, JK, MZ report grant / salary support for unrelated research without conflict of interest. EG, MH, CC, DO, SH, JK, LCB, JvS, SJ, JS, LG report no conflict of interest

MZ reports speaker honoraria at academic institutions. BM reports industry support from Regeneron and Eli Lilly foundation (as investigator, to the institution), MB reports support from the UK National Core Study on Transmission, and PF reports stock in Darvin Biosciences and support (equipment and reagents) from Ceres Nanosciences.

For peer review only - http://bmjopen.bmj.com/site/about/guidelines.xhtml

REFERENCES

1. Ra SH, Lim JS, Kim GU, et al. Upper respiratory viral load in asymptomatic individuals and mildly symptomatic patients with SARS-CoV-2 infection. *Thorax* 2021;76(1):61-63. doi: 10.1136/thoraxjnl-2020-215042 [published Online First: 2020/09/24]
2. Gallichotte EN, Quicke KM, Sexton NR, et al. Early Adoption of Longitudinal Surveillance for SARS-CoV-2 among Staff in Long-Term Care Facilities: Prevalence, Virologic and Sequence Analysis. *Microbiol Spectr* 2021:e0100321. doi: 10.1128/Spectrum.01003-21 [published Online First: 2021/11/11]
3. Gallichotte EN, Quicke KM, Sexton NR, et al. Longitudinal Surveillance for SARS-CoV-2 Among Staff in Six Colorado Long-Term Care Facilities: Epidemiologic, Virologic and Sequence Analysis. *medRxiv* 2020 doi: 10.1101/2020.06.08.20125989 [published Online First: 2020/06/25]
4. Bae SH, Shin H, Koo HY, et al. Asymptomatic Transmission of SARS-CoV-2 on Evacuation Flight. *Emerg Infect Dis* 2020;26(11):2705-08. doi: 10.3201/eid2611.203353 [published Online First: 2020/08/22]
5. Buitrago-Garcia D, Egli-Gany D, Counotte MJ, et al. Occurrence and transmission potential of asymptomatic and presymptomatic SARS-CoV-2 infections: A living systematic review and meta-analysis. *PLoS Med* 2020;17(9):e1003346. doi: 10.1371/journal.pmed.1003346 [published Online First: 2020/09/23]
6. Hart WS, Maini PK, Thompson RN. High infectiousness immediately before COVID-19 symptom onset highlights the importance of continued contact tracing. *Elife* 2021;10 doi: 10.7554/eLife.65534 [published Online First: 2021/04/27]
7. Johansson MA, Quandelacy TM, Kada S, et al. SARS-CoV-2 Transmission From People Without COVID-19 Symptoms. *JAMA Netw Open* 2021;4(1):e2035057. doi: 10.1001/jamanetworkopen.2020.35057 [published Online First: 2021/01/08]
8. Hellewell J, Russell TW, Investigators S, et al. Estimating the effectiveness of routine asymptomatic PCR testing at different frequencies for the detection of SARS-CoV-2 infections. *BMC Med* 2021;19(1):106. doi: 10.1186/s12916-021-01982-x [published Online First: 2021/04/28]
9. Bosch W, Cowart JB, Bhakta S, et al. COVID-19 Vaccine-Breakthrough Infections Requiring Hospitalization in Mayo Clinic Florida through August 2021. *Clin Infect Dis* 2021 doi: 10.1093/cid/ciab932 [published Online First: 2021/11/03]
10. Williams CM, Pan D, Decker J, et al. Exhaled SARS-CoV-2 quantified by face-mask sampling in hospitalised patients with COVID-19. *J Infect* 2021;82(6):253-59. doi: 10.1016/j.jinf.2021.03.018 [published Online First: 2021/03/29]
11. Barer MR. 2020.
12. Nair B. Final Report on the Safety Assessment of Polyvinyl Alcohol. *International Journal of Toxicology* 1998;17(5):67-92. doi: 10.1177/109158189801700505
13. Araujo AM, Petribu AT, Sales Barbosa GH, et al. The use of polyvinyl alcohol glutaraldehyde as solid-phase in ELISA for plague. *Mem Inst Oswaldo Cruz* 1996;91(2):195-8. doi: 10.1590/s0074-02761996000200014 [published Online First: 1996/03/01]
14. Coelho Rde A, Yamasaki H, Perez E, et al. The use of polysiloxane/polyvinyl alcohol beads as solid phase in IgG anti-Toxocara canis detection using a recombinant antigen. *Mem*

For peer review only

For peer review only - http://bmjopen.bmj.com/site/about/guidelines.xhtml

Inst Oswaldo Cruz 2003;98(3):391-3. doi: 10.1590/s0074-02762003000300018 [published Online First: 2003/07/30]

15. Harpaz D, Axelrod T, Yitian AL, et al. Dissolvable Polyvinyl-Alcohol Film, a Time-Barrier to Modulate Sample Flow in a 3D-Printed Holder for Capillary Flow Paper Diagnostics. *Materials (Basel)* 2019;12(3) doi: 10.3390/ma12030343 [published Online First: 2019/01/27]
16. Baker MI, Walsh SP, Schwartz Z, et al. A review of polyvinyl alcohol and its uses in cartilage and orthopedic applications. *J Biomed Mater Res B Appl Biomater* 2012;100(5):1451-7. doi: 10.1002/jbm.b.32694 [published Online First: 2012/04/20]
17. Luo Y, Wu H, Feng C, et al. "One-Pot" Fabrication of Highly Versatile and Biocompatible Poly(vinyl alcohol)-porphyrin-based Nanotheranostics. *Theranostics* 2017;7(16):3901-14. doi: 10.7150/thno.20190 [published Online First: 2017/11/08]
18. Schultz JS, McCarthy MK, Rester C, et al. Development and Validation of a Multiplex Microsphere Immunoassay Using Dried Blood Spots for SARS-CoV-2 Seroprevalence: Application in First Responders in Colorado, USA. *J Clin Microbiol* 2021;59(6) doi: 10.1128/JCM.00290-21 [published Online First: 2021/04/03]
19. Samper ICM, C. J.; Schenkel, M. I.; Clark, K. M.; Khamcharoen, W.; Anderson, L. BR.; Gallichotte, E. N.; Ebel, G. D; Geiss, B. J.; Dandy, D. S.; Henry, C. S. Electrochemical immunoassay for the detection of SARS-CoV-2 nucleocapsid protein in nasopharyngeal samples. *Analytical Chemistry* 2021;Under Review
20. Lendvay TS, Chen J, Harcourt BH, et al. Addressing personal protective equipment (PPE) decontamination: Methylene blue and light inactivates severe acute respiratory coronavirus virus 2 (SARS-CoV-2) on N95 respirators and medical masks with maintenance of integrity and fit. *Infect Control Hosp Epidemiol* 2021:1-10. doi: 10.1017/ice.2021.230 [published Online First: 2021/05/22]

For peer review only

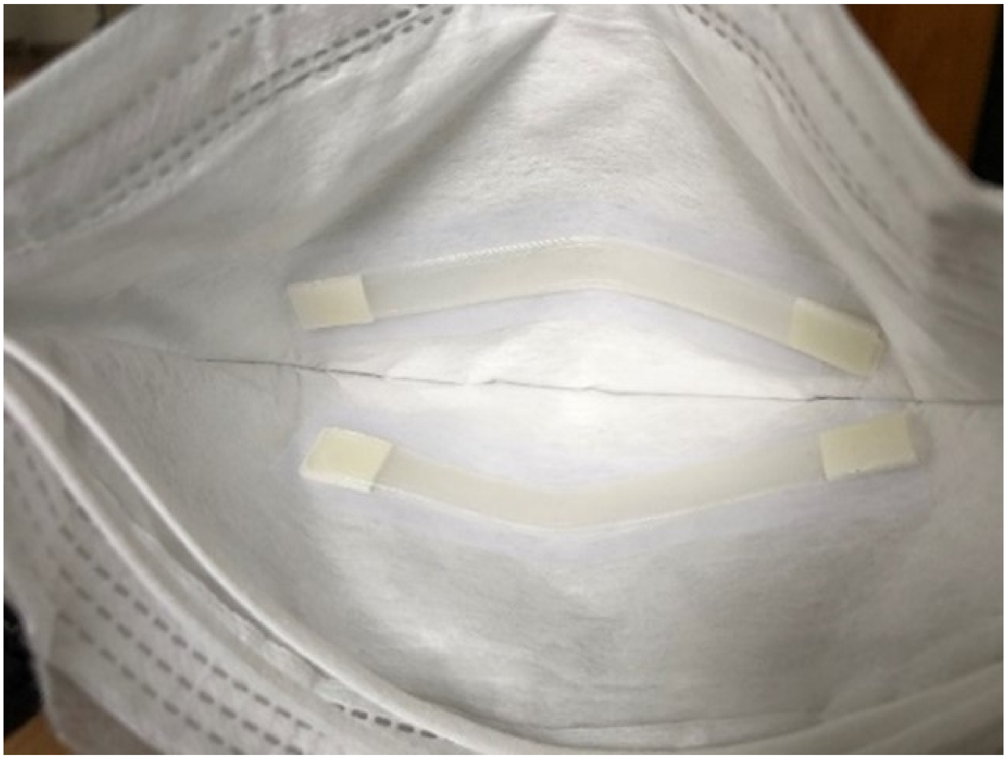

Figure 1: Picture showing two PVA test strips adhered to the inside of a face mask. (Photo credit: Dr. Michael Barer)

73x55mm (220 x 220 DPI)

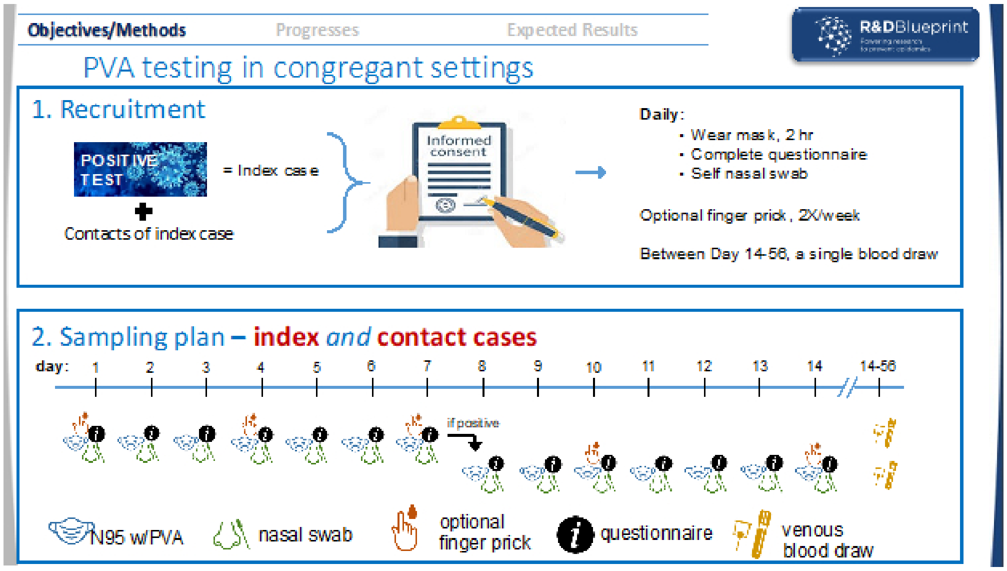

Figure 2. PVA Mask Strip study, sampling plan for index case and contact

417x234mm (38 x 38 DPI)

STROBE Statement—Checklist of items that should be included in reports of ***cohort studies***

	Item No	Recommendation	Page No
**Title and abstract**	1	(*a*) Indicate the study's design with a commonly used term in the title or the abstract	1
		(*b*) Provide in the abstract an informative and balanced summary of what was done and what was found	2
**Introduction**			
Background/rationale	2	Explain the scientific background and rationale for the investigation being reported	3
Objectives	3	State specific objectives, including any prespecified hypotheses	4-6
**Methods**			
Study design	4	Present key elements of study design early in the paper	6-9
Setting	5	Describe the setting, locations, and relevant dates, including periods of recruitment, exposure, follow-up, and data collection	6-9
Participants	6	(*a*) Give the eligibility criteria, and the sources and methods of selection of participants. Describe methods of follow-up	7-8
		(*b*) For matched studies, give matching criteria and number of exposed and unexposed	
Variables	7	Clearly define all outcomes, exposures, predictors, potential confounders, and effect modifiers. Give diagnostic criteria, if applicable	7-8
Data sources/ measurement	8*	For each variable of interest, give sources of data and details of methods of assessment (measurement). Describe comparability of assessment methods if there is more than one group	11-12
Bias	9	Describe any efforts to address potential sources of bias	12
Study size	10	Explain how the study size was arrived at	7
Quantitative variables	11	Explain how quantitative variables were handled in the analyses. If applicable, describe which groupings were chosen and why	N/A
Statistical methods	12	(*a*) Describe all statistical methods, including those used to control for confounding	11-12
		(*b*) Describe any methods used to examine subgroups and interactions	
		(*c*) Explain how missing data were addressed	
		(*d*) If applicable, explain how loss to follow-up was addressed	
		(*e*) Describe any sensitivity analyses	
**Results**			**N/A**
Participants	13*	(a) Report numbers of individuals at each stage of study—eg numbers potentially eligible, examined for eligibility, confirmed eligible, included in the study, completing follow-up, and analysed	
		(b) Give reasons for non-participation at each stage	
		(c) Consider use of a flow diagram	
Descriptive data	14*	(a) Give characteristics of study participants (eg demographic, clinical, social) and information on exposures and potential confounders	
		(b) Indicate number of participants with missing data for each variable of interest	
		(c) Summarise follow-up time (eg, average and total amount)	
Outcome data	15*	Report numbers of outcome events or summary measures over time	

For peer review only

Main results	16	(*a*) Give unadjusted estimates and, if applicable, confounder-adjusted estimates and their precision (eg, 95% confidence interval). Make clear which confounders were adjusted for and why they were included<br>(*b*) Report category boundaries when continuous variables were categorized<br>(*c*) If relevant, consider translating estimates of relative risk into absolute risk for a meaningful time period	
Other analyses	17	Report other analyses done—eg analyses of subgroups and interactions, and sensitivity analyses	
**Discussion**			
Key results	18	Summarise key results with reference to study objectives	N/A
Limitations	19	Discuss limitations of the study, taking into account sources of potential bias or imprecision. Discuss both direction and magnitude of any potential bias	N/A
Interpretation	20	Give a cautious overall interpretation of results considering objectives, limitations, multiplicity of analyses, results from similar studies, and other relevant evidence	N/A
Generalisability	21	Discuss the generalisability (external validity) of the study results	N/A
**Other information**			
Funding	22	Give the source of funding and the role of the funders for the present study and, if applicable, for the original study on which the present article is based	13

*Give information separately for exposed and unexposed groups.

Note: An Explanation and Elaboration article discusses each checklist item and gives methodological background and published examples of transparent reporting. The STROBE checklist is best used in conjunction with this article (freely available on the Web sites of PLoS Medicine at http://www.plosmedicine.org/, Annals of Internal Medicine at http://www.annals.org/, and Epidemiology at http://www.epidem.com/). Information on the STROBE Initiative is available at http://www.strobe-statement.org.

For peer review only

BMJ Open

INVESTIGATING TRANSMISSION OF SARS-COV-2 USING NOVEL FACE MASK SAMPLING: A PROTOCOL FOR AN OBSERVATIONAL PROSPECTIVE STUDY OF INDEX CASES AND THEIR CONTACTS IN A CONGREGATE SETTING

Journal:	*BMJ Open*
Manuscript ID	bmjopen-2022-061029.R1
Article Type:	Protocol
Date Submitted by the Author:	15-Aug-2022
Complete List of Authors:	Jaenisch, Thomas; Colorado School of Public Health, Center for Global Health / Epidemiology; University Hospital Heidelberg, Heidelberg Institute of Global Health<br>Lamb, Molly; Colorado School of Public Health, Center for Global Health / Epidemiology<br>Gallichotte, Emily; Colorado State University, Department of Microbiology, Immunology, and Pathology<br>Adams, Brian; Colorado School of Public Health, Center for Global Health<br>Henry, Charles; Colorado State University, Department of Chemical and Biological Engineering<br>Riess, Jeannine; Colorado State University, Office of Environmental Health Services<br>van Sickle, Joni Triantis; Colorado State University, Office of Environmental Health Services<br>Hawkins, Kellie; Denver Health, Public Health Institute<br>Montague, Brian; University of Colorado, Occupational Health<br>Coburn, Cody; University of Colorado, Occupational Health<br>Conners Bauer, Leisha; University of Colorado Boulder, Health Promotion and Collegiate Recovery Center<br>Kovarik, Jennifer; University of Colorado Boulder, Health Promotion and Collegiate Recovery Center<br>Hernandez, Mark; University of Colorado Boulder, Civil, Environmental and Architectural Engineering<br>Bronson, Amy; Colorado Mesa University, Office of the Vice President<br>Graham, Lucy; Colorado Mesa University, Department of Nursing<br>James, Stephanie ; Regis University, Department of Pharmaceutical Sciences<br>Hanenberg, Stephanie; University of Colorado Colorado Springs, Office of Wellness and Health<br>Kovacs, James; University of Colorado Colorado Springs, Department of Chemistry and Biology<br>Spencer, John; Colorado State University, Department of Microbiology, Immunology, and Pathology<br>Zabel, Mark; Colorado State University, Department of Microbiology, Immunology, and Pathology<br>Fox, Philip; Colorado State University, Department of Microbiology, Immunology, and Pathology<br>Pluss, Olivia; Colorado School of Public Health, Center for Global Health

For peer review only - http://bmjopen.bmj.com/site/about/guidelines.xhtml

	Windsor, William; Colorado School of Public Health, Center for Global Health<br>Winstanley, Geoffrey; Colorado School of Public Health, Center for Global Health<br>Olson, Daniel; Colorado School of Public Health, Center for Global Health<br>Barer, Michael; University of Leicester, Department of Infectious Diseases<br>Berman, Stephen; Colorado School of Public Health, Center for Global Health<br>Ebel, Gregory; Colorado State University, Department of Microbiology, Immunology, and Pathology<br>Chu, May; Colorado School of Public Health, Center for Global Health

**Primary Subject Heading**:	Infectious diseases
Secondary Subject Heading:	Epidemiology
Keywords:	COVID-19, Diagnostic microbiology < INFECTIOUS DISEASES, VIROLOGY

SCHOLARONE™
Manuscripts

TITLE: INVESTIGATING TRANSMISSION OF SARS-COV-2 USING NOVEL FACE MASK SAMPLING: A PROTOCOL FOR AN OBSERVATIONAL PROSPECTIVE STUDY OF INDEX CASES AND THEIR CONTACTS IN A CONGREGATE SETTING

Authors: Thomas Jaenisch[1,2], Molly M. Lamb[1,2], Emily Gallichotte[3], Brian Adams[1], Charles Henry[4], Jeannine Riess[5], Joni Triantis van Sickle[5], Kellie L. Hawkins[6], Brian Montague[6], Cody Coburn[6], Leisha Conners Bauer[8], Jennifer Kovarik[8], Mark Hernandez[9], Amy Bronson[10], Lucy Graham[11], Stephanie James[12], Stephanie Hanenberg[13], James Kovacs[14], John S Spencer[3], Mark Zabel[3], Philip Fox[3], Olivia Pluss[1], William J. Windsor[1], Geoffrey Winstanley[1], Daniel Olson[1], Michael Barer[15], Stephen Berman[1], Gregory Ebel[3], May Chu[1,2]

Affiliations:

[1] Center for Global Health, Colorado School of Public Health, Aurora, CO
[2] Department of Epidemiology, Colorado School of Public Health, Aurora, CO
[3] Department of Microbiology, Immunology, and Pathology, Colorado State University, Fort Collins, CO
[4] Department of Chemical and Biological Engineering, Colorado State University, Fort Collins, CO
[5] Office of Environmental Health Services, Colorado State University, Fort Collins, CO
[6] Public Health Institute at Denver Health, Denver, CO
[7] University of Colorado Anschutz, Occupational Health, Anschutz Medical Center, Aurora, CO
[8] Health Promotion and Collegiate Recovery Center, University of Colorado Boulder, Boulder, CO
[9] Civil, Environmental and Architectural Engineering, University of Colorado Boulder, Boulder CO
[10] Office of the Vice President, Colorado Mesa University, Grand Junction, CO
[11] Department of Nursing, Colorado Mesa University, Grand Junction, CO
[12] Department of Pharmaceutical Sciences, School of Pharmacy, Regis University
[13] Office of Wellness and Health, University of Colorado-Colorado Springs, Colorado Springs, CO
[14] Department of Chemistry and Biology, University of Colorado-Colorado Springs, Colorado Springs, CO
[15] Department of Infectious Diseases, University of Leicester, Leicester, United Kingdom

Corresponding Author:
Thomas Jaenisch, MD PhD
Associate Professor, Center for Global Health & Department of Epidemiology,
Colorado School of Public Health
Aurora, CO 80045
thomas.jaenisch@cuanschutz.edu

Key Words: SARS-CoV-2, transmissibility, transmission, face mask sampling, detection, diagnosis, polyvinyl alcohol, congregate setting, college students, COVID-19, study design

For peer review only - http://bmjopen.bmj.com/site/about/guidelines.xhtml

Abstract:

Introduction: This study aims to measure how transmission of SARS-CoV-2 occurs in communities and to identify conditions that lend to increased transmission focusing on congregate situations. We will measure SARS-CoV-2 in exhaled breath of asymptomatic and symptomatic persons using face mask sampling – a non-invasive method for SARS-CoV-2 detection in exhaled air. We aim to detect transmission clusters and identify risk factors for SARS-CoV-2 transmission in pre-symptomatic, asymptomatic, and symptomatic individuals.

Methods and analysis: In this observational prospective study with daily follow-up, index cases and their respective contacts are identified at each participating institution. Contact definitions are based on CDC and local health department guidelines. Participants will wear masks with polyvinyl alcohol test strips adhered to the inside for 2 hours daily. The strips are applied to all masks used over at least 7 days. In addition, self-administered nasal swabs and (optional) finger prick blood samples are performed by participants. Samples are tested by standard PCR protocols and by novel antigen tests.

Ethics and dissemination: This study was approved by the Colorado Multiple Institutions Review Board (COMIRB) and the World Health Organization Ethics Review Committee and registered with ClinicalTrials.gov (#NCT05145803). From the data generated we will analyze transmission clusters and risk factors for transmission of SARS-CoV-2 in congregate settings. The kinetics of asymptomatic transmission and the evaluation of non-invasive tools for detection of transmissibility are of crucial importance for the development of more targeted control interventions – and ultimately to assist with keeping congregate settings open that are essential for our social fabric.

Strengths and limitations of this study:

- The study employs a novel and non-invasive sampling device
- Following index cases as well as their contacts prospectively enables us to study transmissibility
- Results from the novel sampling device in the face masks are compared with standard nose swab sampling
- The manuscript serves as a template for similar studies to be replicated

For peer review only - http://bmjopen.bmj.com/site/about/guidelines.xhtml

INTRODUCTION

There is an urgent need to better describe and analyze the transmission of SARS-CoV-2 in asymptomatic and pre-symptomatic (infected) individuals, especially regarding the heterogeneity of transmissibility ("super-spreader events"), and within the context of congregate situations. An innovative tool with high sensitivity and minimal invasiveness is needed to obtain the necessary information to fill the current knowledge gaps.

A sizable proportion of individuals infected with SARS-CoV-2 are asymptomatic. These individuals contribute to transmission and are also less likely to get tested. Asymptomatic SARS-CoV-2-infected individuals carry viral loads in conventional samples from the nasopharynx that are in the same range as those of symptomatic individuals, and a significant proportion of SARS-CoV-2 spread is due to asymptomatic individuals [1-6]. An analytical model published by the Centers for Disease Control and Prevention (CDC) found that up to 59% of all transmissions likely resulted from asymptomatic transmission. The model further delineated that 35% of these 59% of infections resulted from those that eventually became symptomatic, while 24% remained asymptomatic [7]. This study also found that the asymptomatic group positive for SARS-CoV-2 was only 25% less infectious compared to those that were symptomatic. This is in contrast to findings from systematic review studies focused on secondary attack rates where the authors concluded that secondar attack rates caused by asymptomatic individuals were only around 1% (and 7% in pre-symptomatic) [8] or within household transmission around 3% (asymptomatic) and 20% (pre-symptomatic) [9]. Overall, few studies were designed specifically to study transmission in congregate situations, including index cases and their close contacts.

With the emergence of virus variants with the potential for greater infectivity, there is also a need to monitor viral variants in congregate situations among asymptomatic individuals, and to rapidly provide new information to public health authorities [10]. This is especially true for breakthrough infections that require special monitoring regarding the details of the vaccines (type, intervals, etc.) as well as the virus variants that caused the breakthrough infection [11].

The reference method for detection of SARS-CoV-2 is the reverse transcriptase polymerase chain reaction (RT-PCR), a technology that detects viral RNA from respiratory specimens (sputum or

For peer review only - http://bmjopen.bmj.com/site/about/guidelines.xhtml

nasal swab) or from saliva. For diagnostic purposes, these samples are usually collected at a single point in time.

To study the dynamics of SARS-CoV-2 transmission over time, we investigate a non-invasive face mask sampling (FMS) method, where polyvinyl alcohol (PVA) strips are embedded in masks for the detection of SARS-CoV-2 in exhaled breath (Figure 1). This enables us to extend the sampling interval over a time window (hours) and include different activities or risk factors such as singing, shouting, sneezing, or coughing as part of the data collection process.

Figure 1: Picture showing two PVA test strips adhered to the inside of a face mask. (Photo credit: Dr. Michael Barer)

The overall aim of this study is to analyze transmission clusters of SARS-CoV-2 in communities and identify factors that may increase transmissibility, with a focus on congregate situations. We will compare the results from the FMS with the standard nose swab. Within sub-studies, we are interested in better understanding antibody profiles and their correlation with symptom status and transmissibility, as well as the effect of different activities (like singing or exercising) on test positivity in the masks. We also plan follow-up studies (which will need additional ethical approval) investigating long-term outcomes in the college student-age population regarding the incidence and the risk factors associated with Long COVID. Details of the study objectives can be found in Box 1.

BOX 1: Objectives

Study Identifier: clinicaltrial.gov identifier NCT05145803

Objective 1: Identification of risk factors for, and rate of, infection in index cases and for transmission from index case to contacts.

Objective 1.1: Describe demographics and evaluate **risk factors for infection** in index cases

Objective 1.2: Evaluate **risk factors for transmission from index case to contacts (and their contacts)**, including viral load, clinical symptoms, vaccination status,

For peer review only

For peer review only - http://bmjopen.bmj.com/site/about/guidelines.xhtml

activity and environmental metrics and immune response profiles (for example neutralizing antibodies, cytokine profiles – using a blood sample taken after recovery)

Objective 1.3: Analyze the **difference in the clinical status (symptomatic vs. asymptomatic)** for index cases as well as secondary cases **by humoral and T-cell immune response profile** (using the blood samples collected).

Objective 1.4: Determine **rate of and time to transmission**, stratified by a variety of risk factors (including presence of clinical symptoms, activity and environmental metrics both in index cases and secondary contacts), analyzing pre-symptomatic and asymptomatic exhalation of SARS-CoV-2 in contacts of index cases.

Objective 2: In confirmed index cases and their contacts, quantify and determine the efficacy of the PVA test strip method for SARS-CoV-2 detection, comparing with the respective 'reference standard' (e.g., nasal swab) collection method, and correlating with the presence of antibodies.

Objective 2.1: Assess the **association between molecular detection by PVA test strip and molecular or antigen detection / quantification** by initial reference diagnostic collection method.

Objective 2.2: Assess the **association between molecular detection by PVA test strip and molecular or antigen detection method** (self-sampling) over the sampling / mask wearing period.

Objective 3: Due to the concern around emerging variants with higher transmissibility, carry out deep sequencing of PCR-positive samples (swabs as well as strips from masks) to derive the genotype of infecting virus.

Objective 3.1: Share in timely fashion the results of the sequencing with the national / regional surveillance system.

Objective 3.2: Assess the **genetic similarities and differences of the SARS-CoV-2 variants** by sequence comparison and by virological culture to assess cell-based infectivity of the strains.

Objective 3.3: Determine the chain of transmission and relationship to clinical phenotype in the index case and contacts where viral RNA or culture permit.

Objective 3.4: Analyze the **difference in rate of transmission** (see also Aim 1) **comparing different SARS-CoV-2 variants**.

For peer review only

For peer review only - http://bmjopen.bmj.com/site/about/guidelines.xhtml

METHODS AND ANALYSIS

Study Design: This is a prospective study with daily follow-up to capture early transmission in the pre-symptomatic phase. Index cases are identified by the routine testing system implemented at each participating institution and contacts of index cases are identified by the site-specific contact tracing system. The contact definition is based on CDC and the local health department guidelines (i.e., individuals who are cohabitating or who were in close [<6 feet], extended [>15 min.] contact with the index case and within 48 hours of diagnosis), and will be updated to reflect the most recent definition available. Study sites have an ongoing active case detection and contact tracing program. Inclusion criteria and endpoints are described in Box 2.

Study population: This study started in the first quarter of 2021 and is currently under way in college students in Colorado, USA, including University of Colorado (Denver, Boulder, Colorado Springs, Aurora), Colorado State University (Fort Collins), Regis University (Denver), as well as Colorado Mesa University (Grand Junction). We envisage enrolment to be completed by April 2022 but would potentially continue beyond this if high SARS-CoV-2 transmission is documented. Students as well as faculty (and their family) are eligible to be enrolled.

The Face Mask Sampling Method: The FMS method has been successfully used to detect Mycobacterium in breath samples and recently adapted for COVID-19 testing [12]. The sensitivity compared to regular nasal swab sampling has not been conclusively determined (which is part of this research project). The (preliminary) analytical sensitivity of this method was estimated around $\geq 10^4$ virus particles, which corresponds to a cycle threshold (Ct) value of 18-20 in the PCR and is in agreement with recent publications and sample testing by other colleagues at the University of Alberta, Canada [13].

Face mask selection: Commercial 3-layer spunbond polypropylene, tested for breathability (95% filtration) KN95 masks that are OSHA/ FDA approved masks will be provided to the participant. Each mask will be uniquely coded for tracking.

For peer review only - http://bmjopen.bmj.com/site/about/guidelines.xhtml

Participant and public involvement: Participants were involved in the selection of best fitting masks in a pilot phase. Feedback was elicited regarding the acceptability and fit of different mask designs.

PVA test strips: PVA test strips are provided by University of Leicester (UL) under a material transfer agreement and strips will be adhered to pre-determined positions inside the face masks. PVA has a very good safety profile, being already used in cosmetic products (face masque) and is approved as hypo-allergenic and non-toxic for humans [14]. PVA has been used in diagnostic tests [15-17] as well as medical device implant for orthopedic surgery [18] and for theranostic nanoplatform therapeutic imagery [19].

Sample Size: This is an exploratory study. We aim for a up to 3,300 individuals, including participants with confirmed SARS-CoV2 infection (index cases) and their contacts. These estimates are based on the conservative estimate of 5% transmission from index cases to a close contact. With a maximum of 300 index cases and ~10 contacts per index case, we estimate that we would detect ~150 secondary cases in contacts.

Box 2: Key Study Design Information

Inclusion / exclusion

Inclusion Criteria:

1. INDEX PATIENTS: Individuals aged ≥ 18 years of any gender tested positive for SARS-CoV-2 by a molecular reference test (PCR test or antigen test).
2. CONTACTS: Individuals aged ≥ 18 years and known to be contacts of the INDEX PATIENTS

Exclusion Criteria: < age 18 years old, unable to wear a face mask due to underlying condition, not able to consent, pregnancy. In addition, signs of severe disease present at time of enrollment (e.g. difficulty breathing, pain when breathing, tightness of chest or developing irregular heartbeat).

Endpoints

A. Outcome Measure(s):

For peer review only - http://bmjopen.bmj.com/site/about/guidelines.xhtml

Objective 1 Outcomes: PVA strip positivity; nose swab test positivity

Objective 2 Outcomes: PVA strip positivity and virus quantification (by PCR); nose swab test positivity; antigen test positivity; presence of antibodies in the blood.

Objective 3 Outcome: Deep sequencing of PCR-positive samples to derive the genotype of the infecting virus.

Schedule of wearing masks: Participants will be asked to wear a mask with 2 PVA test strips adhered to the inside surface for 2 hours per day. Index cases will complete 7 days of mask wearing and if they test positive by the end of the 7-day period, they will be asked to continue for another 7 days up to day 14. Contacts will complete 7 days of mask wearing as long as they test negative. If testing positive in the first 7 days, they will complete an additional 7 days of mask wearing starting with the day when they first test positive. If by the end of this period they still test positive, the duration will be extended by 2-day intervals with their permission until two consecutive masks wear dates test negative (Figure 2). Upon completion of 2 hours of wear by the participant, the face masks will be placed in a sealed plastic bag, collected daily, and brought into the laboratory where the PVA strips will be put into tubes with Viral Transport Medium (VTM). The VTM tubes with the PVA strips will be eluted and tested by PCR.

Self-administered nasal swabs: We ask the participants to collect a self-administered nasal swab daily by placing a nasal swab approximately 0.5 inches into one nostril and swabbing the inside of the nose. The swab is then immediately placed into a 15ml conical tube containing 2ml of VTM. Samples are put into a separate sealed bag and stored at 4°C until testing.

Questionnaires: An electronic questionnaire is administered at the beginning of the mask wearing period to collect basic demographics (age, gender) and to assess COVID-19 symptoms and activities (including the day when symptoms started). During the follow-up period, daily questionnaires are administered, and we have built in warning signs for potential severe COVID-19 that trigger an auto-alert, in which case we will pro-actively contact the participant and a) ask the participant to stop wearing the masks, and b) advise the participant to seek medical care immediately. We also included an optional assessment of activities like singing or exercising for index cases only (confirmed SARS-CoV-2 positive individuals) on one day using a separate

For peer review only - http://bmjopen.bmj.com/site/about/guidelines.xhtml

second mask. In the future, the assessment of vital signs or specific symptoms could be added via wearable technologies or app-based recording (see for example https://www.hyfeapp.com/).

Blood sampling: We will ask the participants to conduct self-sampling of blood using dried blood spots from finger pricks [20] every third day (optional - filter papers and self-sealing bags provided along with the daily masks). Antibodies will be eluted from the filter paper to be used for serological investigations. These samples are going to be stored in separate sealed bags and picked up together with the other materials. After recovery, e.g., between day 14 and 56 after quarantine/self-isolation, the participants are invited to return to the clinic for a venous blood sample (EDTA 20-30 ml). The blood is collected to investigate humoral, and T-cell based immune responses including cytokine profiles and other T cell phenotypic surface markers that may be associated with transmissibility and clinical phenotype (e.g., protection versus Long Covid).

Figure 2. PVA Mask Strip study, sampling plan for index case and contact

Laboratory methods: In a biosafety cabinet, PVA strips are removed from inside of the face masks, by cutting them at the ends where they are adhered using double-sided tape. Each strip is placed into a 15ml conical tube containing 2ml of viral transport media (VTM) and incubated at room temperature for at least 30 minutes to allow PVA to dissolve. Samples are stored at 4°C until testing. After collection and elution of the strip, testing will be carried out with the following tests in order to validate the results from the FMS against the reference test:

a) First line: PCR from the eluate of the strip
b) Second line: antigen test, which gives a positive/negative/indeterminate response
c) Third line: virus culture to determine if virus is infectious.

In brief, for PCR testing, two approaches are used. In the first approach, samples (PVA strips and nasal swabs) are briefly vortexed to thoroughly mix. Viral RNA is extracted from 200ul of sample using the Mag-Bind® Viral DNA/RNA 96 Kit following manufacturers protocol. Quantitative real-team PCR (qRT-PCR) is performed using CDC 2019-nCoV primer probes and EXPRESS qPCR Supermix and SuperScript. Samples are considered positive if their N1 cycle threshold (CT) is less than 38. In the second approach, samples (PVA strips and nasal swabs) are briefly vortexed

For peer review only - http://bmjopen.bmj.com/site/about/guidelines.xhtml

to thoroughly mix. Viral RNA is extracted from 45uL of sample by digesting with 5uL of Proteinase K (PK, NEB) for 15 minutes at 60°C. PK is inactivated for 5 minutes at 95°C. Reverse transcription droplet digital PCR (RT-ddPCR) was performed on PK digested sample using CDC 2019-nCoV primer probes and One-Step RT-ddPCR Advanced Kit for Probes (Bio Rad). Samples are considered positive if their N1 concentration is > 0.25 copies/uL. The RNA extracted from the FMS and potentially recovered live viruses will be used as basis for deep sequencing [3].

One of our aims is to validate a novel antigen assay for testing PVA strips from the masks. For this assay, electrodes were prepared according to Samper et al. [21]. For sample analysis, 20 µL of PVA mask sample or virus standard was added to the functionalized electrode and incubated for 40 min, and then rinsed with PBST and PBS using solid stream spray bottles. Virus standards were inactivated with 0.1% Igepal and diluted in Hanks Balanced Buffer Solution (HBSS) with 0.1% Tween80 and 0.1% Igepal. Following sample incubation, 20 µL of a 0.5 µg/mL HRP-antibody solution was added and incubated for 25 min, and then rinsed with PBST and PBS using solid stream spray bottles. For measurement, 50 µL of 3,3',5,5'-tetramethylbenzidine (TMB) was added to the electrode and incubated for 2 min. After 2 min, chronoamperometry was run at 0.0 V for 2 min.

Contactless delivery and pick-up of supplies: All masks and other samples are delivered / picked up at the residence of the participants and placed in their own sealed bag with a unique identifier. All exposed masks and collected sample kits are individually packaged compliant with UN3314 packaging safety requirements. For the decontamination of surfaces and PPE, we will use 70% alcohol for immediate decontamination of exposed surfaces, especially those that the participant may have touched. For longer term decontamination, we will use 10 uM methylene blue spray that allows for up to 10 hours of decontamination in light [22]. Hand hygiene solutions will be used to clean hands at the end of each drop-off/pick up activity.

Data management: All study data will be collected using Research Electronic Data Capture (REDCap®). Data cleaning and quality control assessments will be conducted on the REDCap survey data, and a weekly data dashboard will be produced to support the study team in detecting issues with data collection. Indicators of severe disease will be monitored daily. Test results from

For peer review only - http://bmjopen.bmj.com/site/about/guidelines.xhtml

the reference test, the additional molecular tests, antibody tests, and the PVA strip test will be linked to survey data using a unique anonymized study identification number, and data will be stored on a secure internal server with restricted access at the University of Colorado with no personal identifiers. The study analyst will hold the key to the personal identifiers and will unblind results as necessary to inform the institution of any previously unknown positive cases, so that they can follow the institution's protocols regarding informing the case, contact tracing, and quarantine. All data will be cleaned, merged and analyzed using SAS version 9.4 (Cary, NC), to ensure replicability of the process and analysis.

Data analysis: For objective 1, we will first conduct a descriptive analysis of the index cases. Then we will conduct a risk factor analysis using multivariable multi-level regression models to determine symptoms and behaviors that are associated with greater exhalation of SARS-CoV_2 virus, as detected by PVA test strips. We will also analyze presence and timing of symptoms in contacts to determine if PVA strips can detect SARS-CoV-2 in exhaled breath of pre-symptomatic and asymptomatic individuals. We will map the transmission dynamics visually, determine the length of exhalation of SARS-CoV-2 in cases, and calculate the difference in rate of transmission by vaccination status. Using the convalescent blood sample, we will analyze different immune response profiles (neutralizing antibodies, T-cell based assays) stratified by clinical phenotype (symptomatic/asymptomatic, potentially using a symptom score for increased granularity).

To address objective 2, we will calculate the sensitivity and specificity of the FMS method, using the initial reference diagnostic method of the site in index cases. We will then calculate the sensitivity and specificity of the PVA test strips using the concurrently collected nasal swab as the reference standard for both index cases and their contacts. We will also correlate strip test positivity with Ct value of the PCR test (or viral load in a subset) over time and with presence of antibodies (IgA and IgG) over time.

To address the concern about emergence of new virus variants, in objective 3, the FMS eluted materials, original specimen and/or recovered live virus isolates will be subjected to deep sequencing to monitor genetic variants in the study population. We will use these data, when available, to calculate the infectiousness of each viral variant, and to map the transmission chains and association with disease in contacts. We will provide the sequence information of identified SARS-CoV-2 variants (and aliquot of material) to national and global data warehouses for public health surveillance of virus variation that may impact disease transmission and resistance to

For peer review only - http://bmjopen.bmj.com/site/about/guidelines.xhtml

vaccines. The remaining materials will be stored for longer term so that they can be accessed for public health surveillance purposes.

In addition, the samples and data generated in this study will provide ample opportunity for laboratory analysis such as immune profiling or viral genome copy quantification and sequencing. Specific analyses will depend on the experiments and number of confirmed cases - however, will likely include ANOVA, student t-test, and other analyses. Laboratory data will be examined for normality of distribution as necessary, and the appropriate parametric or non-parametric descriptive analyses will be conducted to determine significant differences.

ETHICS AND DISSEMINATION

Ethical approval for a multicentric study in adults (college students and faculty) was obtained by the Colorado Multiple Institutional Review Board (COMIRB) at Anschutz Medical Campus in Aurora (#21-2823) and by the World Health Organization Ethics Review Committee (CERC.0096). In addition, ethical approval for a pilot study in middle schools (not described in detail in this manuscript) was obtained by the Colorado Medical Institutional Review Board (COMIRB, #21-4944) and an adapted protocol for elementary schools is prepared. Here we will concentrate on the study in the adult population.

There is only minimal risk to the participant associated with this study. Discomfort may arise form wearing masks for extended periods of time or from additional reference validation tests (optional, nasal swabs, sputum tests and/or blood samples), though these are routine procedures for SARS-CoV-2 protection, testing and surveillance in the partnering sites / institutions. For validation purposes, participants will also carry out self-sampling of the anterior nose, which is a minimal risk procedure, and does not cause a lot of inconvenience (different from the naso-pharyngeal sampling). For comparison purposes, participants may also be asked to provide a saliva sample, which is not associated with any risk and minimal inconvenience. One venous blood sample (20-30ml) will be taken after recovery to investigate humoral, and T-cell based immune responses, including cytokine profiles and other T cell phenotypic surface markers that may be associated with transmissibility and clinical phenotype (protection versus long term Covid). This is a minimal risk procedure, carried out by professionals. Inconvenience includes possible bruising and some

For peer review only - http://bmjopen.bmj.com/site/about/guidelines.xhtml

pain. Finger prick blood samples are optional and are considered minimal risk. They might be inconvenient, and we will let participants decide about participating in this.

We present this study design as a tool to further investigate the transmissibility of SARS-CoV-2. We want to share our scientific approach implemented in institutes of higher education which have routine COVID-19 monitoring in place. We would be happy to help replicating this study in other settings worldwide. This study will help determine transmission chains and risk factors for transmission of SARS-CoV-2 in congregate settings. PVA test strips, a non-invasive method, are investigated as a novel and promising tool to detect exhalation of SARS-CoV-2 from people who are actively infected as well as their exposed contacts.

FUNDING STATEMENT

The authors acknowledge that funds for the project were provided by WHO based on a grant from the German Federal Ministry of Health (BMG).

AUTHORS CONTRIBUTIONS

TJ, MML and MC conceptualized the study. MC acquired funding. MB developed the face mask sampling method. MML and WJW developed the data base, curate the data, and developed the data analysis approach. MC, GE, EG, CH, MH, JSS, MZ, JK, PF carry out lab investigations and developed the laboratory testing methodology. SB, TJ, GW, BA, KLH, JvS, JR, BM, CC, LCB, JK, AB, LG, SJ, SH, OP, WJW, DO developed the participant recruitment strategy and conducted the recruitment. TJ and MC wrote the first draft. All authors contributed to the writing of the manuscript and approved the final version.

COMPETING INTERESTS STATEMENT

TJ, MML, JW, BA, GE, MC report grant support from WHO for this study for salaries or equipment/reagents (see also acknowledgements). SB, OP, WJW, AB, CH, JR, JK, MZ report grant / salary support for unrelated research without conflict of interest. EG, MH, CC, DO, SH, JK, LCB, JvS, SJ, JSS, LG report no conflict of interest.

MZ reports speaker honoraria at academic institutions. BM reports industry support from Regeneron and Eli Lilly foundation (as investigator, to the institution), MB reports support from

For peer review only

For peer review only - http://bmjopen.bmj.com/site/about/guidelines.xhtml

the UK National Core Study on Transmission, and PF reports stock in Darvin Biosciences and support (equipment and reagents) from Ceres Nanosciences.

For peer review only

For peer review only - http://bmjopen.bmj.com/site/about/guidelines.xhtml

REFERENCES

1. Ra SH, Lim JS, Kim GU, et al. Upper respiratory viral load in asymptomatic individuals and mildly symptomatic patients with SARS-CoV-2 infection. *Thorax* 2021;76(1):61-63. doi: 10.1136/thoraxjnl-2020-215042 [published Online First: 2020/09/24]

2. Gallichotte EN, Quicke KM, Sexton NR, et al. Early Adoption of Longitudinal Surveillance for SARS-CoV-2 among Staff in Long-Term Care Facilities: Prevalence, Virologic and Sequence Analysis. *Microbiol Spectr* 2021:e0100321. doi: 10.1128/Spectrum.01003-21 [published Online First: 2021/11/11]

3. Gallichotte EN, Quicke KM, Sexton NR, et al. Longitudinal Surveillance for SARS-CoV-2 Among Staff in Six Colorado Long-Term Care Facilities: Epidemiologic, Virologic and Sequence Analysis. *medRxiv* 2020 doi: 10.1101/2020.06.08.20125989 [published Online First: 2020/06/25]

4. Bae SH, Shin H, Koo HY, et al. Asymptomatic Transmission of SARS-CoV-2 on Evacuation Flight. *Emerg Infect Dis* 2020;26(11):2705-08. doi: 10.3201/eid2611.203353 [published Online First: 2020/08/22]

5. Buitrago-Garcia D, Egli-Gany D, Counotte MJ, et al. Occurrence and transmission potential of asymptomatic and presymptomatic SARS-CoV-2 infections: A living systematic review and meta-analysis. *PLoS Med* 2020;17(9):e1003346. doi: 10.1371/journal.pmed.1003346 [published Online First: 2020/09/23]

6. Hart WS, Maini PK, Thompson RN. High infectiousness immediately before COVID-19 symptom onset highlights the importance of continued contact tracing. *Elife* 2021;10 doi: 10.7554/eLife.65534 [published Online First: 2021/04/27]

7. Johansson MA, Quandelacy TM, Kada S, et al. SARS-CoV-2 Transmission From People Without COVID-19 Symptoms. *JAMA Netw Open* 2021;4(1):e2035057. doi: 10.1001/jamanetworkopen.2020.35057 [published Online First: 2021/01/08]

8. Qiu X, Nergiz AI, Maraolo AE, et al. The role of asymptomatic and pre-symptomatic infection in SARS-CoV-2 transmission-a living systematic review. *Clin Microbiol Infect* 2021;27(4):511-19. doi: 10.1016/j.cmi.2021.01.011 [published Online First: 2021/01/24]

9. Madewell ZJ, Yang Y, Longini IM, Jr., et al. Factors Associated With Household Transmission of SARS-CoV-2: An Updated Systematic Review and Meta-analysis. *JAMA Netw Open* 2021;4(8):e2122240. doi: 10.1001/jamanetworkopen.2021.22240 [published Online First: 2021/08/28]

10. Hellewell J, Russell TW, Investigators S, et al. Estimating the effectiveness of routine asymptomatic PCR testing at different frequencies for the detection of SARS-CoV-2 infections. *BMC Med* 2021;19(1):106. doi: 10.1186/s12916-021-01982-x [published Online First: 2021/04/28]

11. Bosch W, Cowart JB, Bhakta S, et al. COVID-19 Vaccine-Breakthrough Infections Requiring Hospitalization in Mayo Clinic Florida through August 2021. *Clin Infect Dis* 2021 doi: 10.1093/cid/ciab932 [published Online First: 2021/11/03]

12. Williams CM, Pan D, Decker J, et al. Exhaled SARS-CoV-2 quantified by face-mask sampling in hospitalised patients with COVID-19. *J Infect* 2021;82(6):253-59. doi: 10.1016/j.jinf.2021.03.018 [published Online First: 2021/03/29]

13. Barer MR. 2020.

14. Nair B. Final Report on the Safety Assessment of Polyvinyl Alcohol. *International Journal of Toxicology* 1998;17(5):67-92. doi: 10.1177/109158189801700505

For peer review only - http://bmjopen.bmj.com/site/about/guidelines.xhtml

15. Araujo AM, Petribu AT, Sales Barbosa GH, et al. The use of polyvinyl alcohol glutaraldehyde as solid-phase in ELISA for plague. *Mem Inst Oswaldo Cruz* 1996;91(2):195-8. doi: 10.1590/s0074-02761996000200014 [published Online First: 1996/03/01]

16. Coelho Rde A, Yamasaki H, Perez E, et al. The use of polysiloxane/polyvinyl alcohol beads as solid phase in IgG anti-Toxocara canis detection using a recombinant antigen. *Mem Inst Oswaldo Cruz* 2003;98(3):391-3. doi: 10.1590/s0074-02762003000300018 [published Online First: 2003/07/30]

17. Harpaz D, Axelrod T, Yitian AL, et al. Dissolvable Polyvinyl-Alcohol Film, a Time-Barrier to Modulate Sample Flow in a 3D-Printed Holder for Capillary Flow Paper Diagnostics. *Materials (Basel)* 2019;12(3) doi: 10.3390/ma12030343 [published Online First: 2019/01/27]

18. Baker MI, Walsh SP, Schwartz Z, et al. A review of polyvinyl alcohol and its uses in cartilage and orthopedic applications. *J Biomed Mater Res B Appl Biomater* 2012;100(5):1451-7. doi: 10.1002/jbm.b.32694 [published Online First: 2012/04/20]

19. Luo Y, Wu H, Feng C, et al. "One-Pot" Fabrication of Highly Versatile and Biocompatible Poly(vinyl alcohol)-porphyrin-based Nanotheranostics. *Theranostics* 2017;7(16):3901-14. doi: 10.7150/thno.20190 [published Online First: 2017/11/08]

20. Schultz JS, McCarthy MK, Rester C, et al. Development and Validation of a Multiplex Microsphere Immunoassay Using Dried Blood Spots for SARS-CoV-2 Seroprevalence: Application in First Responders in Colorado, USA. *J Clin Microbiol* 2021;59(6) doi: 10.1128/JCM.00290-21 [published Online First: 2021/04/03]

21. Samper ICM, C. J.; Schenkel, M. I.; Clark, K. M.; Khamcharoen, W.; Anderson, L. BR.; Gallichotte, E. N.; Ebel, G. D; Geiss, B. J.; Dandy, D. S.; Henry, C. S. Electrochemical immunoassay for the detection of SARS-CoV-2 nucleocapsid protein in nasopharyngeal samples. *Analytical Chemistry* 2021;Under Review

22. Lendvay TS, Chen J, Harcourt BH, et al. Addressing personal protective equipment (PPE) decontamination: Methylene blue and light inactivates severe acute respiratory coronavirus virus 2 (SARS-CoV-2) on N95 respirators and medical masks with maintenance of integrity and fit. *Infect Control Hosp Epidemiol* 2021:1-10. doi: 10.1017/ice.2021.230 [published Online First: 2021/05/22]

For peer review only

For peer review only - http://bmjopen.bmj.com/site/about/guidelines.xhtml

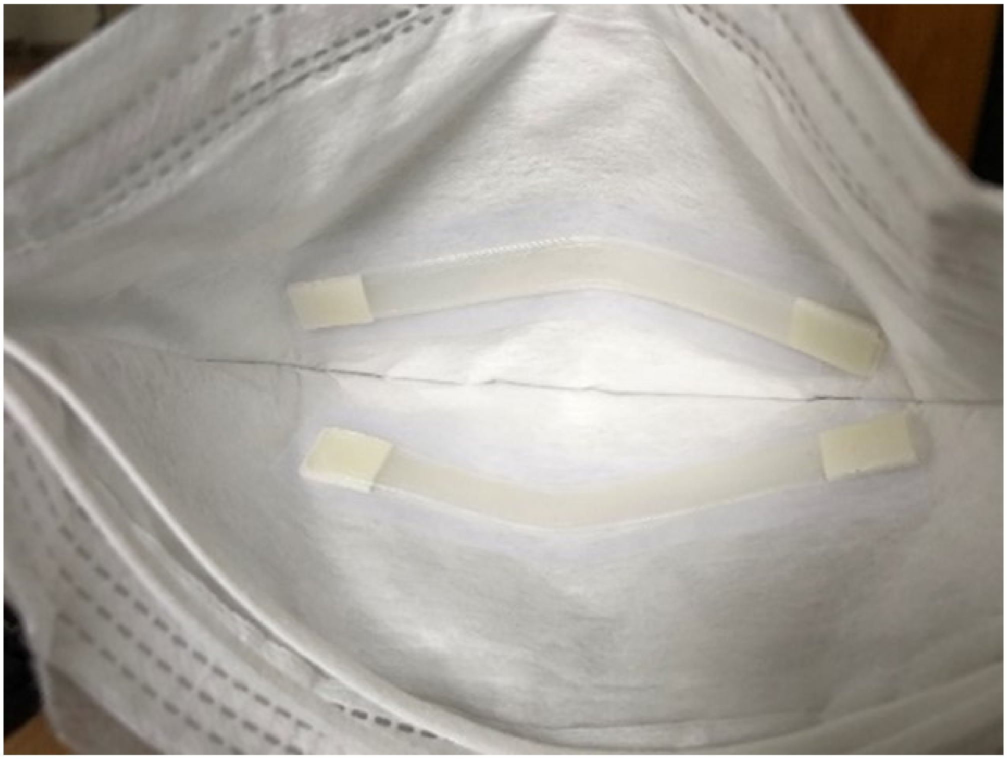

Figure 1: Picture showing two PVA test strips adhered to the inside of a face mask. (Photo credit: Dr. Michael Barer)

73x55mm (220 x 220 DPI)

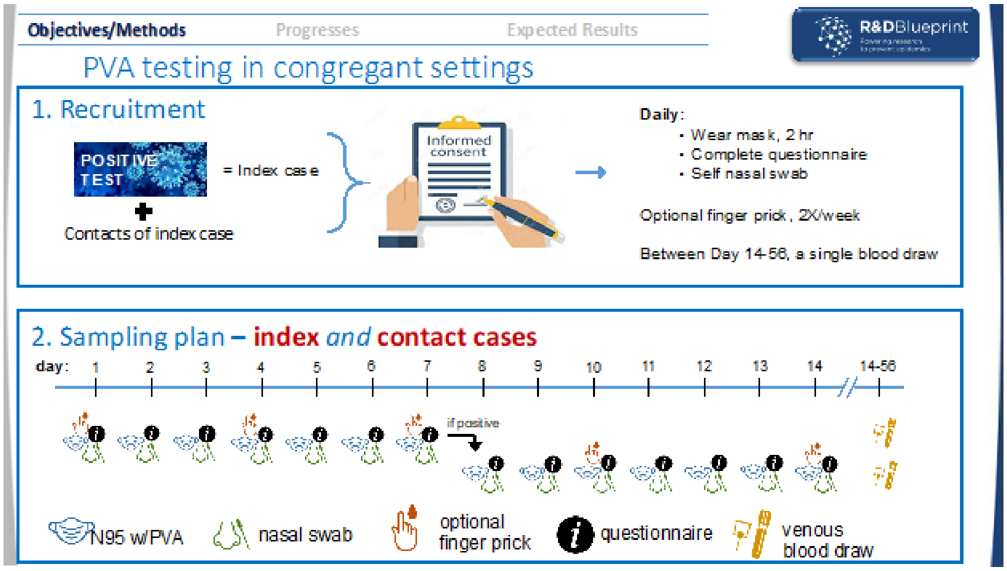

Figure 2. PVA Mask Strip study, sampling plan for index case and contact

417x234mm (38 x 38 DPI)
